# Endovascular aneurysm repair in emergent ruptured abdominal aortic aneurysm with a ‘real’ hostile neck and severely tortuous iliac artery of an elderly patient

**DOI:** 10.1186/1471-2482-14-11

**Published:** 2014-03-05

**Authors:** Nan Wu, Changwei Liu, Qining Fu, Rong Zeng, Yu Chen, Genhuan Yang, Bao Liu

**Affiliations:** 1 Department of surgery, Peking Union Medical College Hospital, Peking Union Medical College and Chinese Academy of Medical Sciences, Beijing 100730, P.R. China; 2 Department of vascular surgery, Peking Union Medical College Hospital, Peking Union Medical College and Chinese Academy of Medical Sciences, No. 1 Shuaifuyuan, Beijing 100730, P.R. China; 3 Department of vascular surgery, The first Affiliated Hospital of Chongqing Medical University, Chongqing 400016, P.R. China

**Keywords:** Endovascular aneurysm repair (EVAR), Ruptured abdominal aortic aneurysm (rAAA), Hostile anatomy, Elderly patient

## Abstract

**Background:**

Endovascular aneurysm repair (EVAR) has been a revolutionary development in the treatment of abdominal aortic aneurysms (AAAs). Meanwhile, unfavorable anatomy of the aneurysm has always been a challenge to vascular surgeons, and the application of EVAR in emergent and elderly patients are still in dispute.

**Case presentation:**

A 79-year-old woman presented as an emergency of abdominal pain with acute hypotension, heart rate elevation and a rapid decrease of hemoglobin. Emergent computed tomographic angiography (CTA) showed a ruptured AAA (rAAA) extending from below the opening of bilateral renal arteries down to the celiac artery and elongated to both common iliac arteries. The hostile neck and severely tortuous iliac artery made the following procedure a great challenge. An emergent endovascular approach was performed in which an excluder aortic main body was deployed below the origin of the bilateral renal arteries covering the ruptured aortic segment. Two iliac legs were placed superior to the opening of the right hypogastric respectively. In order to avoid the type Ib endoleak, we tried to deploy another cuff above the bifurcation of the iliac artery. However, the severely tortuous right iliac artery made this procedure extremely difficult, and a balloon-assisted technique was used in order to keep the stiff wire stable. Another iliac leg was placed above the bifurcation of the left iliac artery. The following angiography showed a severe Ia endoleak in the proximal neck and therefore, a cuff was deployed distal to opening of the left renal artery with off-the-shelf solution. The patient had an uneventful recovery with a resolution of the rAAA. She is well and symptom-free 6 months later.

**Conclusion:**

Endovascular aneurysm repair (EVAR) in emergent elderly rAAA with hostile neck and severe tortuous iliac arter**y** is extremely challenging, and endovascular management with integrated technique is feasible and may achieve a satisfactory early result.

## Background

Endovascular aneurysm repair (EVAR) has been a revolutionary development in the treatment of abdominal aortic aneurysms (AAAs). The advent of endovascular technology and the increasing experience and expertise of endovascular specialists have had a profound impact on the management of aortic aneurysms and resulted in improved perioperative outcomes and late results comparable with conventional open surgical repair [1-3]. However, unfavorable morphology of the aneurysm and adverse anatomic characteristics of the aortic neck in particular have restricted the widespread applicability of EVAR [4-6]. Besides, the application of EVAR in both emergent and elderly patients has been controversial [7].

Herein, we report EVAR in an emergent ruptured AAA (rAAA) of an elderly patient who was with hostile neck and severely tortuous iliac artery.

## Case presentation

**Figure 1:**
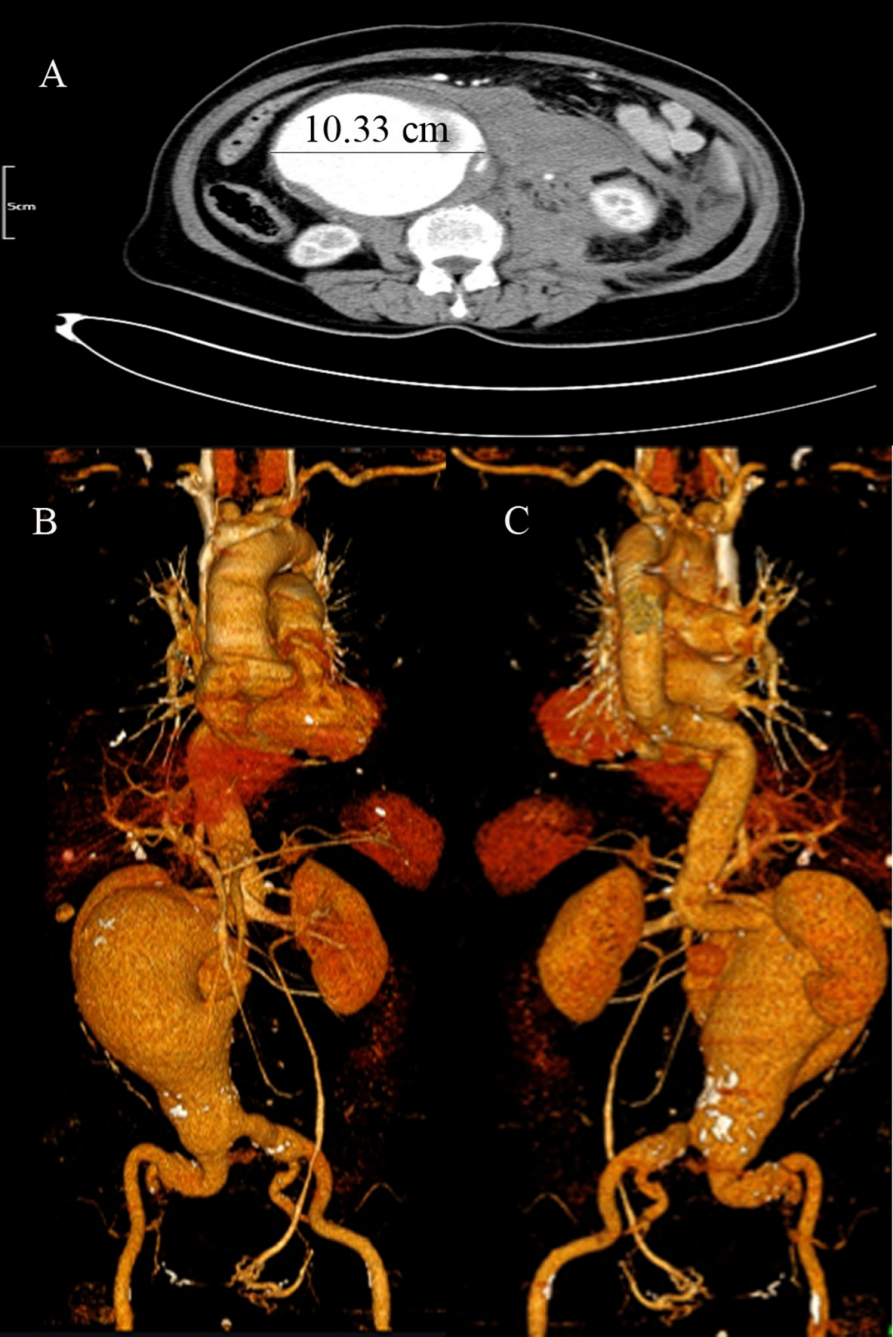
**Preoperative CTA and 3-dimensional reconstructions. (A)** Preoperative CTA shows a ruptured AAA with a maximum diameter of 10.33 cm in axial view. **(B, C)** 3-dimensional reconstructions confirmed the rupture extended from 2.5 cm below the level of right renal artery down to the celiac artery. A sever tortuous right celiac artery was observed with a ‘S’ appearance.

A 79-year-old woman was admitted to the emergency department of Peking Union Medical College Hospital complaining of severe abdominal pain with unknown causes for 4 hours. The pain got worse after 2 hours with an acute hypotension (blood pressure drop from 106/79 mmHg to 61/44 mmHg), heart rate elevation (from 80 beats/min to 100 beats/min) and blunted. Laboratory investigation noted a decrease of hemoglobin from 119 g/dL to 62 g/dL in half an hour. Emergent computed tomographic angiography (CTA) showed the presence of a very tortuously ruptured AAA that now was 10.33 cm in diameter with a 90° neck angle (Figure 1A). The rupture, which extended from 2.5 cm below the opening of bilateral renal arteries down to the celiac artery and elongated to both common iliac arteries, involved the inferior mesenteric artery. Although the two iliac arteries were unobstructed with a 2.1 cm and 1.8 cm diameter of the right and left respectively, the right iliac artery was severely tortuous with a ‘S’ appearance (Figure 1B and C).

**Figure 2:**
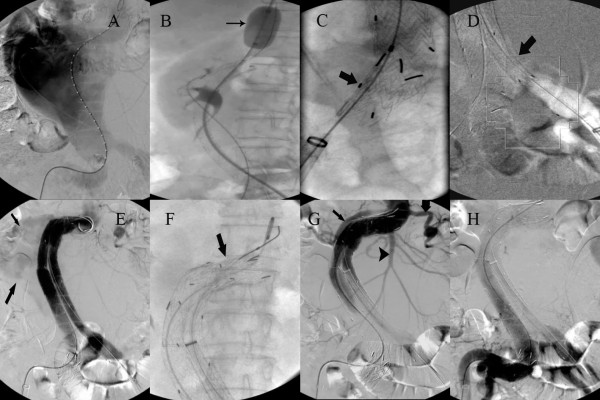
**The angiography during the operation. (A)** Diagnostic angiography showing the presence of a voluminous AAA. **(B)** Two iliac legs placing superior to the opening of the right hypogastric artery with balloon-assisted technique (arrow shows the balloon) **(C)** Another cuff deploying above the bifurcation of the iliac artery to avoid the type Ib endoleak. **(D)** An iliac leg placing above the bifurcation of the left iliac artery. **(E)** The following angiography showing a severe Ia endoleak in the proximal neck. **(F)** A Cuff deploying distal to opening of the left renal artery. **(G and H)** Final angiography confirming the successful sealing of rAAA (thin arrow and the thick arrow show the complete right and left renal artery respectively, arrowhead shows the superior mesenteric artery).

Previous medical history was normal except that she had a hypertension for more than 10 years with a maximum blood pressure of 160/90 mmHg, and was diagnosed of AAA by CTA with a maximum diameter of 6 cm in 2009. She refused to follow up because of no abdominal symptoms since then. She had no surgical history. Given the age of the patient and the emergent status, an endovascular approach was conducted despite the hostile neck. The time of door-to-cath lab was about 70 minutes. After local anesthesia, a 6-F, 11 cm-long introducer sheath (Cordis, Johnson & Johnson, NJ, USA) was placed percutaneously through the left groin for the diagnostic angiography, which confirmed the existence of an unfavorable aneurysm inferior to the renal artery (Figure 2A and Additional file 1: Movie S1). An 18-F, 30-cm-long sheath (W.L. Gore & Associates, Flagstaff, AZ, USA) was introduced and a 28-12-160-mm Excluder aortic main body (W.L. Gore &Associates) was deployed below the origin of the bilateral renal arteries (Additional file 2: Movie S2). Another 18-F, 30-cm-long sheath (W.L. Gore & Associates, Flagstaff, AZ, USA) was used, and two iliac legs of 20–120 and 20–100 (W.L. Gore & Associates, Flagstaff, AZ, USA) were placed superior to the opening of the right hypogastric respectively.

The guidewire was hard to enter the iliac leg due to the severely tortuous right iliac artery (Additional file 3: Movie S3), and therefore balloon-assisted technique was designed. The balloon was deployed in the proximal part of the main body and then inflated, thus keep the stiff wire in place which allowing the positioning of the guidewire (Figure 2B and Additional file 4: Movie S4).

In order to avoid the type Ib endoleak, we deployed a 26–30 cuff above the bifurcation of the iliac artery (W.L. Gore & Associates, Flagstaff, AZ, USA) (Figure 2C). A 20–140 iliac leg was placed above the bifurcation of the left iliac artery (Figure 2D). The stent-grafts were dilated using balloons (CODA, COOK, USA). The following angiography showed a severe Ia endoleak in the proximal neck (Figure 2E and Additional file 5: Movie S5), and therefore, a 32–4.0 Cuff (W.L. Gore & Associates, Flagstaff, AZ, USA) was deployed distal to opening of the left renal artery with off-the-shelf solution [8] (Figure 2F).

**Figure 3:**
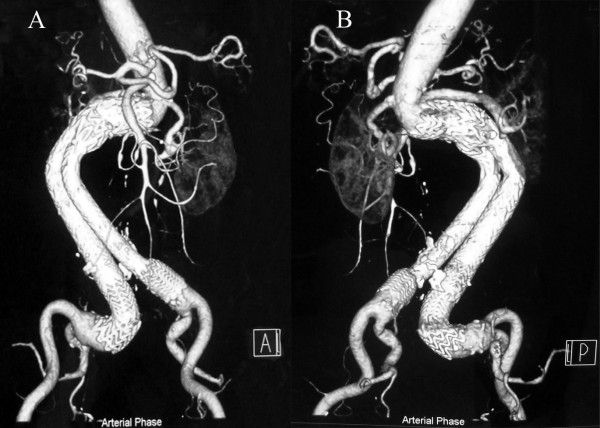
Anterior (A) and posterior (B) view of CT reconstruction at 6-month follow-up shows fluent celiac and bilateral renal arteries.

Final angiography confirmed successful sealing of the rAAA with continued perfusion of both celiac and right renal arteries, and the aortic body pulsation disappeared accordingly (Figure 2G, H and Additional file 6: Movie S6). The patient had an uneventful recovery and was discharged home after 6 days. She is well and symptom-free 6 months later. Follow-up CT at 6 months demonstrated fluent celiac and bilateral renal arteries (Figure 3).

## Conclusion

Although EVAR has been performed since 1990s [9], anatomical factors such as unfavorable neck due to angulation, morphology, or calcification are still a challenge for vascular surgeons [2]. In this case, the hostile neck and the tortuous iliac artery made her unfavorable for EVAR. However, in consideration of the need of rapid intervention and a rather older age, an endovascular approach was performed.

### EVAR in patients with hostile neck anatomy

Argues exist in the efficacy of EVAR in patients with hostile neck anatomy. Hostile neck was defined as conditions that were not consistent with the instructions for use of endograft devices employed in the selected studies. In one study, the neck length < 15 mm, neck angulation > 60 degrees and proximal neck thrombus or calcification covering > 50% circumference of the aortic diameter and a reverse taper morphology were considered as hostile neck anatomy [4]. The meta-analysis enrolled seven studies compared the outcomes of EVAR in patients with hostile and friendly neck anatomy. They found that, in technically, patients with hostile anatomy required an increased number of adjunctive procedures to achieve proximal seal compared with friendly ones. The immediate and early outcome showed that although no significant differences in the incidence of type I endoleak and reintervention rates within 30 days of treatment was identified between the EVAR and open surgery groups, patients with unfavorable neck anatomy suffered from higher risk of 30-day morbidity (OR, 2.278; 95% CI, 1.025-5.063). In the one year follow up, higher risk of type I endoleak and aneurysm-related mortality were noticed in hostile anatomy patients and a nine fold increased risk of aneurysm-related mortality within 1 year of treatment. They came to the conclusion that EVAR should be used cautiously in patients with hostile neck anatomy [4]. The data from European Collaborators on Stent-Graft Techniques for Aortic Aneurysm Repair database showed a significantly increased risk of proximal endoleaks at short-term and medium-term follow-up after EVAR in patients with short (< 15 mm) proximal neck. Severe (> 60 degrees) infrarenal aortic neck angulation was found to be associated with higher incidences of proximal neck dilation, proximal type I endoleak, and need for secondary interventions [5,6]. Stather et al. systemically reviewed the outcomes following EVAR in patients with hostile neck anatomy (HNA) and those with favorable neck anatomy, they suggested that EVAR in patients with HNA may accompanied with increased technical difficulty and a worse short-term outcomes. Besides, they found higher rates of early and late type I endoleaks, along with secondary interventions in HNA patients and recommended that increased monitoring should be performed in these patients [10].

In this case, we performed EVAR despite the unfavorable hostile neck with a 90° angle and the severely tortuous iliac artery. Additional cuff was placed so as to avoid the type Ib endoleak after deploying of two iliac legs above the right iliac artery. It has been very difficult to finish this complex procedure in such a tortuous iliac artery.

The balloon-assisted technique designed here provided efficient positioning of the stiff wire, which stablly facilitated the advancement of the guide wire. This approach has been used when advancing a sheath hindered by a tortuous or angulated course. Prieto et al. described a similar balloon-assisted technique using a balloon inflated in a distal pulmonary artery branch to better anchor the wire while advancing the sheath through a tortuous anatomy [11]. Peeling et al. reported their experience of balloon-assisted technique to overcome extreme tortuous cervical carotid. They found that hypercompliant balloon catheters can be reliably used to facilitate safe and rapid distal positioning of guiding catheters beyond severe cervical tortuosity [12]. This technique enabled us efficiently advance guidewire through the unfriendly anatomy, thus decreasing operation time and radiation exposure. Meanwhile, this could also help us to avoid undesirable injuries to vessels when toughly introducing the stiff sheath. Of course, besides the strategy we used, several other maneuvers might be adopted in that particular situation. For examples, we could catch the wire by snares through a brachial artery puncture. In addition, the wire could also be fixed through the two directions of brachial artery and humeral artery. However, the risk of aortic dissection was accordingly increased. Because the elderly patient usually accompanied with a tortuous aorta or angulated artery of distal part from subclavian artery, it is probable to damage the initial part of descending aorta when fixing the wire.

Despite the rising prevalence of endoleak with the increasing number of EVAR, endoleak management continues to be a challenge [13]. In this particular case, additional cuff was deployed after the implantation of aortic main body and two iliac legs in order to avoid the Ib endoleak. Besides, a severe Ia endoleak was noted intraoperatively, therefore we deployed another cuff distal to the opening of the left renal artery to seal the endoleak. It was reported that the more unfavorable an AAA neck anatomy is the higher incidence of complications such as endoleak is accompanied with [14]. Fortunately, the use of off-the-shelf technique for endoleak management has been proven to be reliable [8,15]. For example, Perdikides et al. [16] reported 13 AAA patients with unfavorable neck performing EVAR, two proximal Ia endoleaks were detected after completion angiography, and a cuff was deployed in one of them which successfully sealed the endoleak. Although no endoleak was found after 3 months follow up in this case, life-long follow up is still needed.

Advancing sheath through such an unfavorable anatomy can be a challenge to even the most experienced interventionalist. During EVAR procedure, we tried to advance the catheter into the common iliac artery, but this proved very difficult because the severely tortuous right iliac artery resulting in repeated kinking of the catheter. Therefore, we proposed a balloon-assisted technique that better anchored the wire while advancing the sheath through the tortuous path which eased guidewire engagement. The balloon supported and guided the stiff wire by temporarily obstructing the aorta. With the balloon inflated and anchored distally, stiff wire could successfully pass through the angulated artery while maintaining distal wire position. Once the sheath is advanced across the unfavorable anatomy, the distal “anchoring balloon” was deflated and withdrawn over the guidewire without loss of wire position.

### EVAR in ruptured AAA

Multiple reports have showed that EVAR for rAAA has become accepted as a viable treatment. In a recent retrospective observational study involving 338278 patients, Schermerhorn et al. [7] demonstrated that a reduction in overall rupture repair mortality was noticed after the introduction of EVAR for both ruptured and intact AAA. Another study from Mehta reported a single-center experience comparing EVAR and open surgery for rAAA. Over a 9-year period, EVAR was performed in 120 patients and open surgery in 163 patients. The 30-day mortality for EVAR was 24.2% while a 44.2% for open surgery (*P* < 0.005). Furthermore, 5-year survival was better in the EVAR group (37 vs 26%, *P* < 0.005). The data also showed a shift of clinical practice principle in the treatment of rAAA [17]. Mayer et al. [18] examined all rAAA at two large European institutions, and found a statistical decrease in mortality of patients undergoing EVAR for rAAA compared to open surgery. Interestingly, there were differences in the analysis based on whether patients undergoing EVAR needed further abdominal decompression for compartment syndrome. Increased mortality was found in patients do not need abdominal decompression in comparison with EVAR (odds ratio = 5.6), while no such difference of mortality was observed in patients with abdominal decompression. These results demonstrated that EVAR in patients with symptomatic aneurysms or contained ruptures may achieve a better prognosis than those with frank rupture. Another systemic analysis of eleven studies involving 42,888 patients showed that EVAR was associated with significantly lower mortality than open surgery. Besides, this difference remained when the population-based data were examined separately [19].

As a rapid developing technique, the demerits of EVAR in rAAA can not be ignored. Despite of the technical development in EVAR, rAAA is still a difficult challenge especially in patients with hostile anatomy and unstable hemodynamics. Hoornweg et al. [20] reviewed 83 rAAA patients in which 45 (54.2%) were unsuitable for EVAR because of the anatomy such as unfavorable infrarenal neck or iliac arteries. While EVAR may be feasible for rAAA repairment, randomizing patients to EVAR or open surgery for rAAA was hard to perform. In one trial from UK, the researchers were only able to enroll 32 of 103 patients who were admitted with suspected rAAA. There were no differences in 30-day mortality and complications between EVAR and open surgery [21]. Even though accumulating data showing the reduced mortality of EVAR in rAAA, concern exists that this may reflect a selection bias. It has been suggested that only the most stable patients with ruptures are chosen for EVAR, and the most common reason for open repair was hemodynamic instability preoperatively [21-23]. In a review of discharge data from four states, researchers found that lower-risk patients were selected to perform EVAR compared with those undergo open surgery [24].Furthermore, it should also be noticed that these promising data of EVAR in rAAA were mostly provided by experienced surgeons of famous centers. Additional observation is needed after translating this technique to small or rural hospitals.

### EVAR in the elderly patients

The benefit of a prophylactic procedure in the most elderly AAA patients is unproven [7]. Initial thoughts behind EVAR assumed that it would be more advantageous in the elderly and in those patients unfit for open repair. This debate continues these years. Schermerhorn et al. [25] demonstrated that although operative mortality increases with age, a low mortality rate can be achieved even in Medicare patients of 85 years and older, informing that EVAR in older patients can achieve a better result. Another investigation found that EVAR can be performed safely in octogenarians, despite of the higher American Society of Anesthesiologists classification, larger aneurysms, and more tortuous anatomy, which inducing the longer hospital stay and surgical operating times in older group [26]. Meanwhile, some authors gave an unclear point of view on EVAR in elderly patient. de Blic et al., evaluated the short-term to midterm results after AAA repair in patients >85 years old from a single center. In conclusion, they suggested that elective repair may be proposed in these elderly patients in cases of threatening AAA. Although no significant benefits were noted in EVAR group, it was reasonable to perform this less invasive procedure in elderly patients with suitable anatomies [27]. On the other hand, the famous EVAR 1 trial reported that the older patients had more complications than the ‘fit’ patients [2,28]. Moreover, the Standard Open Surgery Versus Endovascular Repair of Abdominal Aortic Aneurysm (OVER) trial showed that patients over 70 years old who received open surgery tended to have lower mortality than those who were performed with EVAR [3,29]. We suggest that EVAR might be feasible for those elderly patients who are hemodynamically stable or urgent for intervention, of course more cautions should be given to patients with hostile anatomies.

All in all, EVAR is a safe and efficacious treatment for AAA even in complicated cases. EVAR in rAAA has been shown to have improved long-term survival in certain studies. While perioperative mortality is lower with EVAR, long-term outcomes are similar between EVAR and open repair, including quality of life and cost-effectiveness. Further randomized studies comparing EVAR and open surgery for AAA are being needed to adequately make sure which is superior in the long-term results.

### Consent

Written informed consent was obtained from the patient for publication of this case report including associated images and video.

## Abbreviations

EVAR: Endovascular aneurysm repair; AAAs: Abdominal aortic aneurysms; rAAA: ruptured abdominal aortic aneurysm; CTA: Computed tomographic angiography.

## Competing interests

The authors declare that they have no competing interests.

## Authors’ contributions

All authors were involved in the preparation of this manuscript. NW performed the operation, collected the data and wrote the manuscript. CL performed the operation and designed the study. QF summarized the data and revised the manuscript. RZ, YC, GY performed the operation and collected the data. BL made substantial contribution to the study design, performed the operation and revised the manuscript. All authors read and approved the final manuscript.

## Pre-publication history

The pre-publication history for this paper can be accessed here:

http://www.biomedcentral.com/1471-2482/14/11/prepub

## Supplementary Material

Additional file 1: Movie S1 The shape of the guidewire reflected the severely tortuous aneurysm. Click here for file

Additional file 2: Movie S2 Deploying the aortic main body. Click here for file

Additional file 3: Movie S3 The guidewire was hard to enter the iliac leg. Click here for file

Additional file 4: Movie S4 Balloon assisted technique. Click here for file

Additional file 5: Movie S5 The following angiography showing a severe Ia endoleak. Click here for file

Additional file 6: Movie S6 Final angiography. Click here for file
